# Implementation of pharmaceutical care for older adults in the brazilian public health system: a case study and realistic evaluation

**DOI:** 10.1186/s12913-020-4898-z

**Published:** 2020-01-14

**Authors:** Barbara Barros Silva, Claudia Fegadolli

**Affiliations:** Unifesp – Federal University of São Paulo, Institute of Environmental, Chemical and Pharmaceutical Sciences, Street São Nicolau, n 210 - Centro, Diadema, SP CEP: 09913-030 Brazil

**Keywords:** Pharmaceutical care, Organizational innovation, Evaluation, Patient care team

## Abstract

**Background:**

Pharmaceutical care services have been recognized as the most highly regarded professional pharmacy practice model that allows the identification, intervention, and resolution of drug related problems. This practice provides significant clinical outcomes and can reduce direct and indirect costs for health systems. However, its implementation can be complex and challenging, needing study experiences that aims at overcoming obstacles, especially in free and universal healthcare systems. The objective of this study is to evaluate the implementation of Ambulatory Care Pharmacy services for older adults at Paulista Institute of Geriatrics and Gerontology (IPGG), which is recognized in the city of São Paulo for offering pharmaceutical care services for over 10 years continuously. This initiative and process is independent of external academic interventions or educational institutions. It is hoped that the results may also contribute to advancing the implementation of pharmaceutical care service in similar health systems.

**Design:**

This is a case study using multiple sources of data. Qualitative and quantitative data were collected from institutional documents, by participant observation and interviews. Initial themes were identified by content analysis and analyzed under the context-mechanism-outcome configurations (CMO Configurations) in realistic evaluation.

**Setting:**

Geriatrics and Gerontology Institute of São Paulo (known as IPGG).

**Participants:**

Eleven health professionals and three pharmaceutical care service users.

**Results:**

Three CMO configurations were identified and accepted: “Scenario Construction mediated by educational processes”, “Contribution to complex needs resolution”, and “Organizational Visibility”. The CMO (Context-Mechanism-Outcomes) configuration “Logistic activities discourage clinical pharmaceutical services implantation” was denied due to the influence of accepted CMOs.

**Conclusions:**

Educational processes which value transdisciplinary knowledge exchanges provide resources required to overcome important obstacles present during pharmaceutical care implementation. Thus, providing and seeking knowledge to build and offer context-consistent clinical health services as well as fulfilling organizational environment requirements can be the key to implement pharmaceutical care service.

## Background

Drug-related problems cost approximately $42 billion a year worldwide [1] and can be better managed and reduced by pharmaceutical care implementation in health care services [2–5]. Clinical Pharmaceutical services prevent adverse reactions and hospitalizations by decreasing drug-related morbidity [2] and they can improve quality of life, especially in older adult patients with chronic conditions [6]. Moreover, diagnostic exams, hospitalizations, consultations, visits to other health services and medication costs are potentially decreased [7, 8].

The decrease in expenses resulting from pharmaceutical care implementation can generate savings of US$ 5377 per adverse event avoided [5] and $421,810 per year per pharmacist carrying out clinical practice [9] . Strand and Cipolle (2004) identified a decrease of $1,134,162 in total expenditures over 3 years [8].

Systematic review and meta-analysis studies show significant results in terms of economic benefit, hospital admissions, length of hospital stay, mortality and resolution of drug-related problems, but indicate that such results are possible and conclusive only when clinical pharmaceutical services adopt systematic analysis of pharmacotherapy [2, 10–12]. It is evident that, when patients are systematically monitored by a pharmacist, they may exhibit better results related to blood pressure, glycated hemoglobin, albuminuria, renal failure and hyperlipidemia, including polymedicated elderly patients [13].

The pharmacist’s role in promoting health as a member of the health care team has mainly been developed from Mikeal’s ideas (1975) and later by Strand and Hepler’s (1990), as a result the pharmacist’ duty of today are increasingly more patient-orientated since the primary focus of the pharmacist is the patients’ well-being [14, 15]. These principles resulted in the design of a new professional practice called Pharmaceutical Care [15], which assumes that knowledge of patients’ health needs is a priority during the treatment of patients [15, 16]. Since the 1990s, this idea has mainly been driven by the World Health Organization, Pan American Health Organization and by the International Pharmaceutical Federation, influencing worldwide organization of pharmaceutical services with different degrees of implementation among countries [17].

In providing pharmaceutical care using a systematic approach, pharmacists work with the patient in an individualized way using crucial stages such as the initial evaluation, identification of drug-related problem, scheduling and accompaniment of complementary actions (i.e. educational actions) [16]. The PW (pharmacotherapy workup) method, one of the most used methods worldwide, was developed in the United States, where the health system is dominated by insurance companies with little state participation [18].

In contrast, health care in Brazil is mostly offered by a national health system, called *SUS (Sistema Único de Saúde)*, a state system that serves about 70% of the population and is complemented by supplementary health care and direct out-of-pocket cost [19]. In this context, it is complex to implement this practice model (pharmacotherapy workup) designed in the American system, with individualized and compensated actions in a country with a mixed health system, large state responsibility and high population demand with limited public resources [18],

Contrary to what happens in United States of America (USA) pharmacies, in Brazilian public pharmacies, pharmacists are responsible for the dispensing of unpaid drugs to the population and perform a very challenging work related to the logistical management of the drugs, especially regarding the planning, supply and resolution of problems related to the lack of drugs. In addition to this aspect of care, professionals do not usually have additional time or receive additional payment for performing clinical activities [19–21]. This scenario submits to the possibility of professionals assuming new roles only when a favorable interface is established in relation to management, micropolitics, and the ability of learning and execution of clinical activities so that they can overcome implementation barriers and maintain the same previously determined attributions.

In Brazil, there has been a State Pharmaceutical Assistance Program since 1971, and since 1988 the integral right to health including medicines became constitutional, introducing to this program other actions for the benefit of the health of the population [22]. Since then, new policies and rules have been developed as an incentive to incorporate pharmaceutical services, including the pharmaceutical care into public and private health services [22, 23]. In addition to the conceptual and regulatory bases, Brazil’s Health Ministry started to qualify pharmaceutical care actions by the SUS National Qualification Program of State Pharmaceutical Assistance Program (known as *Qualifar-SUS*) and has invested in pharmaceutical care service implementation programs [24]. Thereafter, transformation initiatives take place, however, these services begin normally, but do not continue because a majority of them are not implemented in a strategic manner, but with little planning and systematization of processes and results [25–30]. However, systematization is essential in the implementation of pharmaceutical services, as evaluated by Gil and collaborators (2013), in Spain, and Sorensen and cols (2016), in the USA, as well as Feletto and collaborators (2013) and Moullin and collaborators (2016) in Australia [31–34].

In Brazil, there are gaps in scientific knowledge that make it difficult to understand the slowness of adopting pharmaceutical care in health services, since there are few implementation initiative studies about this kind of practice [32, 34, 35]. Although many limits and challenges in implementing pharmaceutical care services in Brazil are already relatively known [36, 37], not much has been studied from the service organization perspective, the possibility of overcoming obstacles and implementing this professional practice model in Brazilian health services. Currently, most of the implementation experiences occur in partnerships with universities, making it impossible to elucidate the daily reality in services where these partnerships are not present or are not the main organization responsible for structuring actions of implementation [38].

However, new services or technology implementations in a real context is a complex process, due to the countless variables influencing human relationships as well as the various dimensions of humans needs [39, 40]. Making changes in this environment is a challenge and requires implementation strategies using evidence based on practice [39]. In this regard, a study that elucidates how Brazilian pharmaceutical services can be structured to overcome challenges and to obtain, to a certain extent, benefits demonstrated in experimental designs can subsidize new actions in the management and promotion of health education. Thus, the objective of this study is to evaluate the implementation of Ambulatory Care Pharmacy services for older adult patients at Paulista Institute of Geriatrics and Gerontology (IPGG), which is recognized in the city of São Paulo for offering pharmaceutical care services for over 10 years continuously.

## Methods

This is a case study that allows the understanding of complex, real and contemporary phenomena through the study of multiple sources of evidence [41]. The main analysis unit (case) was the implementation of the pharmaceutical care at the Geriatrics and Gerontology Institute of São Paulo. This Institute has approximately 104,000 older adult patients registered, not hospitalized, with an average age of 69 years and registers an average of 40,000 visits per month of several professionals, including two pharmacists. Local pharmacists community, in São Paulo, recognized the pharmaceutical service as a successful implementation model of pharmaceutical care. An important implementation fact is that initially it occurred independently, without the support of research centers or universities, which made the authors of this study to perceive that the pharmacists’ strategies can possibly be replicated in other services in similar contexts and whose implementation does not receive external support.

At the study site, the main professional activities of pharmacists are the logistical management of drugs, which include selection, planning, storage control and care actions, such as dispensing, patient education and support to the multi-professional team to solve drug related problems. Concerning professional support, pharmacists assist patients referred by other professionals and discuss cases with the health team. They also prepare the team of assistants to identify patients who may be facing problems with their medicines and, in these cases, the patients are referred to individual pharmaceutical care.

The exploratory phase included researcher insertion into the search field, study preparation and adaptation between August 2015 and August 2016. Data were collected between August 30, 2016 and June 30, 2017 and data analysis was performed from December 2017 to August 2018.The data related to patient assisted and clinical service were retrospectively collected referring to 2 years (August 2013 to August 2015). The dataset was composed of triangulation data for validation and reliability.

### Identifying principles and theory that drove the implementation of pharmaceutical care

Firstly, the researchers confirmed the theory basing the implementation of Pharmaceutical care in IPGG. According to institutional documents and preliminary interviews, the developer pharmacist focused on implementing the service based on individual care models, using hybrid form templates to ensure consultation such as the Dáder and PW method [15, 16, 42]. As part of their responsibilities, pharmacists hoped to produce individual pharmaceutical consultations using standardized methods and instruments to solve drug-related problems. They also planned to evaluate the number of interventions performed and number and type of solved problems related to medicines. Although the main mission of pharmacists under the vision of IPGG’ managers was to resolve issues related to access to medicines, pharmacists had a clear idea that the logistic management would be the element that would enable the implementation of pharmaceutical care, that is, their focus was on patients. So, we can identify complexity related to differences in expectations or possible contradictions between theory and practice, added to doubts about the applicability of models imported from health systems constituted in different scenarios. This confirms the adequacy of the theory to the analysis of the implementation of pharmaceutical care in IPGG. This approach has the advantage of valuing the context in the understanding of the real life functioning of planned actions.

Then, a realistic evaluation was performed according to Pawson and Tilley principles [43] with thematic analysis of the collected material. The objective was to framing theories to explain implementation process, originating hypothesis and using Context-mechanism-outcomes configuration hypothesis to test theory for refinement. Thus, all data collection was directed by the researchers’ focus on the context, mechanism and results. They included:

- Document analysis

Internal documents of the pharmaceutical care service and the IPGG were analyzed, including productivity reports, presentation and disclosure materials, normative operational documents, research production, patient records, and communication reports of pharmacists with other professionals.

- Participant observation

The participant observation was performed until obtaining data saturation to report professional attitudes, physical and structural characteristics, roles played by workers, customer demands and service operation. To obtain more reliable data, the observers came to the study site on several days of the week as well as several periods to monitor the time organization of the pharmacists and periods of higher and lower demand for service. The relevant aspects of the observation, based on the criteria mentioned, were recorded in the field diary.

- Individual interviews and focus group

The participants of interviews and focal group were selected using snowball and the selection criteria regarding the number of pharmaceutical appointments respectively, and they were invited by telephone. Thirty patients with the largest number of pharmaceutical appointments were selected and were invited to the focus group.

The focus group’s script included questions about the services provided by the pharmacy, about pharmaceutical care, about the type of support sought in the pharmacy, were the difficulties and gains, besides relationship with patients, team professionals and management and about how patients evaluate the quality of service offered by the pharmacy. The professionals of the multi-professional team were interviewed individually and the interview script included questions about the knowledge on pharmaceutical care service. Interviewees were encouraged to describe the functioning of the service, to express opinions on the contribution of pharmacists to health care promotion at the IPGG and to explain how they interact with pharmacists. The interviews with the pharmacists were guided by questions about the origin of this service and how it is conducted.

The individual interviews and focal group discussion were conducted in IPGG and even though guided by a semi-structured script. It was developed for this study and it was a preliminar guide, that allowed that new questions emerged due to the manifestations of respondents and participants. An additional file shows more detail [see Additional file 1].

### Identifying implementation components

To carry out the thematic analysis, all interviews and focal groups were transcribed. The thematic analysis of the collected material (individual interviews, focus group, field diary and document analysis) was performed including the pre-analysis stages, identification of theme indicators, definition of the themes and grouping of themes [37]. Organization and data analysis were carried out using Nvivo® software version 10 produced by QSR International (“NVivo qualitative data analysis software | QSR International [44, 45].

The identified themes were analyzed with respect to their coherence and articulation with the categories provided for in the theoretical and analytical framework of the realistic assessment (Context, Mechanism and Results).

#### Building CMO hypotheses and theories

Contexts (C), mechanisms (M) and outcomes (O) involved in the present phenomenon were identified in order to interrogate what works for whom in what circumstances during the service implementation [43, 46, 47]. It was assumed that mechanisms are resources influenced by the context to produce logic used, and consequently determine people’s reactions to produce results through the implementation of interventions [45]. Mechanisms are triggers and levers which logical processes exert a force that in turns produce results in that context [43]. In contexts existing before implementation, mechanisms cannot be triggered intentionally [47], however, mechanisms enable interventions (intentional).

CMOs configurations (relationships between contexts, mechanisms, and results) were constructed, analyzed and defined to explain and determine factors present during the IPGG pharmaceutical care service implementation process [43, 47]. All analysis required several discussion and reflection sessions between two researchers (BBS and CF).

## Results

### Composition of documentary analysis material, interviews and focus group

Two hundred ninety internal documents were analyzed corresponding to pharmaceutical care service and Institute’s organization, which include: 60 Productivity reports (Indicators, strategic planning, reports for managers, feedback for staff and institutional goals), 38 presentation and disclosure materials, 15 standard operating procedure documents, 23 research documents and symposium awards, 224 medical records, and 129 reports on reporting conduct to other professionals.

In addition, 1080 h of participant observation were performed, a focus group (2 h duration) involving the participation of three pharmaceutical care service users and 11 in-depth interviews (30 min to 1 h duration) with health professionals were performed. The health professionals who were invited and participated were members of the multi-professional team which includes a manager of pharmaceutical services coordinator, IPGG pharmaceutical services director, a pharmacist, a speech therapist, a nurse, a psychologist, a social worker and two physicians. Of the 11 health professionals included, 10 were female and one of them was a male.

Concerning the information related to patients, the 224 medical records analyzed corresponded to the total number of patients seen at the clinical pharmaceutical service in the selected period. The mean age of patients was 76 years, 98.8% were aged between 60 and 92 years, 94.18% between 65 and 87 years, 75% over 69 years and 50% between 69 and 83 years. The majority (72%) of the patients were women. These resulted in the production of 861 pharmaceutical consultations, with a mean of 4 consultations per patient during the period studied. It was possible to identify the detailed list of medications of 79 patients, with an average of 10 medications per patient. For the other patients, these lists were not fully available or updated in medical records, so they were not included in the count.

Of the 30 patients initially included for participation in the focus group, it was possible to contact and invite by phone 20 patients, no one refused, however, only 3 attended. Refusals and drop-outs were due to the fact that most phones are not up to date in local databases, difficulty in communicating by telephone with the elderly, distance from the clinic of attendance, transportation difficulties and forgetting the date and time. The three participants that attended were women aged 86, 72 and 70.

### Identifying theory

The first stage of the thematic analysis identified the following thematic indicators in Nvivo: Institutional policy, Characteristics of the population attended to, perspective of those involved on pharmaceutical services, adaptation of structural conditions, internal and external communication, replanning and construction of indicators. These themes were later grouped and discussed in depth into 3 categories: work context, mechanisms on viability, and institutionalization of pharmaceutical care. The articulated analysis of these categories and themes subsidized the construction of 4 CMOs.

**Fig. 1:**
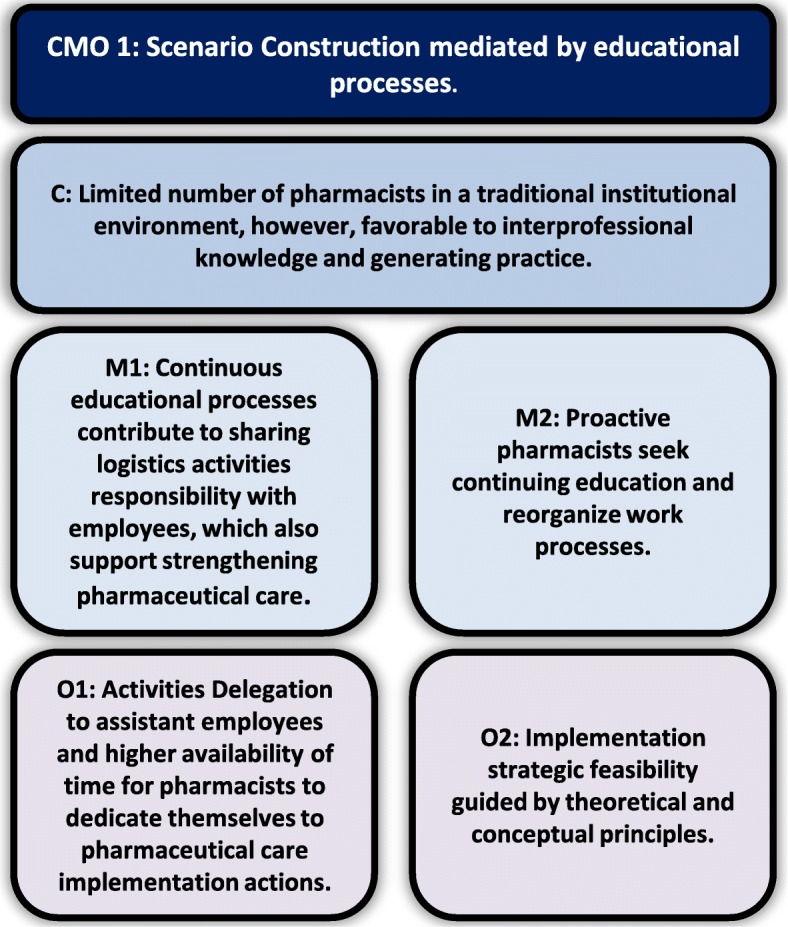
Inter-relationship between C (Context), M (Mechanism) and O (Outcomes) of scenario construction mediated by educational processes

**Fig. 2:**
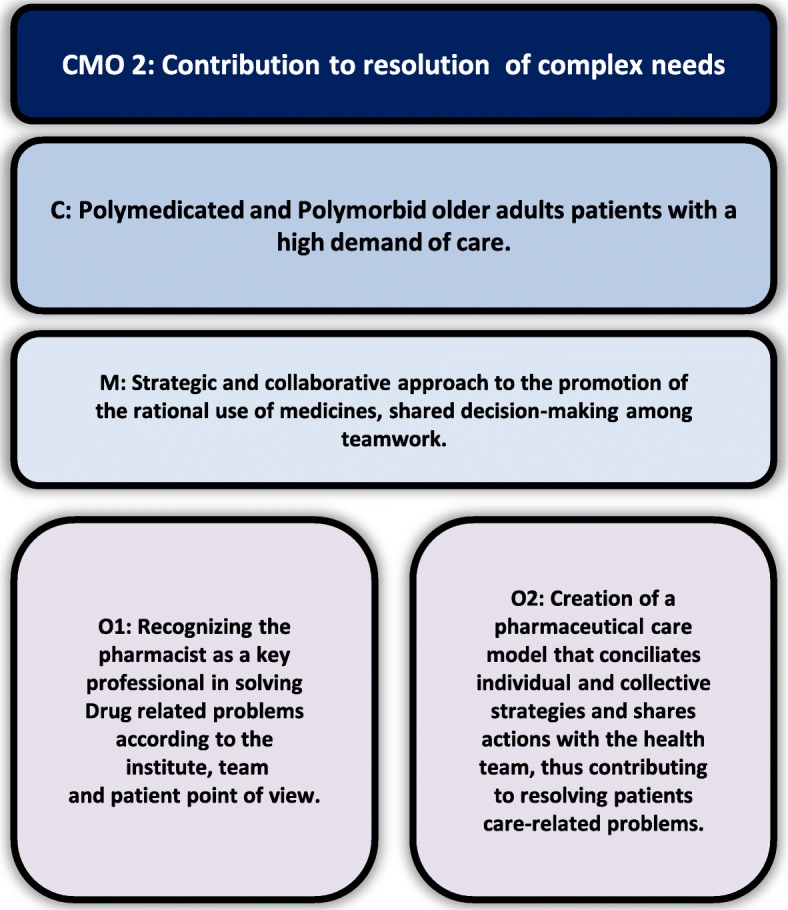
Inter-relationship between C (Context), M (Mechanism) and O (Outcomes) of Contribution to resolution of complex needs

**Fig. 3:**
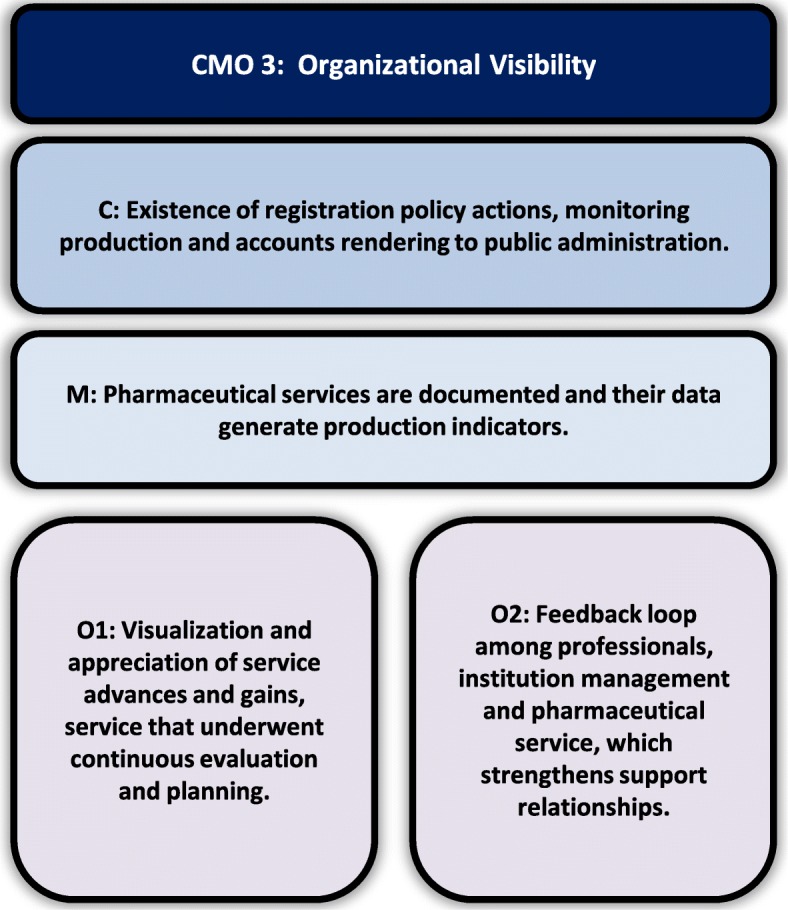
Inter-relationship between C (Context), M (Mechanism) and O (Outcomes) of organizational visibility

Data analysis, hypothesis construction and test of theories resulted in three CMO configurations that explain the implementation of pharmaceutical care at IPGG: Scenario Construction mediated by educational processes (Fig. 1), Contribution to resolution of complex needs (Fig. 2), and Organizational Visibility (Fig. 3).

#### Scenario construction mediated by educational processes

Although the number of IPGG pharmacists is reduced to perform all the activities traditionally attributed to these professionals, it was possible to develop mechanisms to overcome this limitation, mainly due to the favorable context of learning and adopting new roles. IPGG stimulates interaction and knowledge exchange among interdisciplinary team professionals. The favorable organizational tendency to interprofessional knowledge development, educational meetings and training of all involved in the institute positively influences the pharmaceutical care implementation process. The positioning of the institute’s management board illustrates this:

*“We are a center that promotes health education for the elderly, so we receive researchers, build research (...) we are a participating center that works to train a network.” (Management board of IPGG).*

The creation of the implementation processes is favorable, resistance to organizational change is diminished and the multi-professional team becomes an ally to pharmaceutical care execution when the interprofessional knowledge are constructed (pharmacists, assistants and others professionals). Auxiliary employees were included in the course of the pharmaceutical care process, assuming the role of identifying patients with specific care needs during medication dispensing. They received training to identify priority patients for individual care, such as those who have little understanding about the disease and treatment, who have polypharmacy or access difficulties. Consequently, they became responsible for 40% of the pharmaceutical care referral service.

This number indicates that the time of dispensing is valued as an opportunity to identify problems, in which assistants play a key role. The number of patients attended monthly by these assistants, in the study period, approximately 2839, favors the participation of these workers in the pharmaceutical care process. This quantity is higher than the number of consultations of the medical team, for example, which attends approximately 1100 patients per month, in the study period. In addition, during the pharmacists’ working hours, cases are discussed with the multi-professional team, especially doctors, without formal referral, scheduling or documentation, which reduces the need for referral by these professionals.

*Excerpts from a talk with the pharmacists and the field diary illustrate this process:*

*“So, we have strategic people who support the flow of this work inclusively even when we are not here”. (Pharmacist2).*

*“They can identify patients’ needs at the time of dispensing drugs, they were trained so that, when they identify an elderly with a greater drug related problem, they call us, or if we are in a meeting or we are absent, they make referrals to pharmaceutical care service. (Pharmacist 1).*

*“The positive point here is that all drug dispensations are properly performed by the employee resulting in patient’s guidance. The patient tries to talk about his experiences subjective to medication use, such as fear and expectations, but the employee is limited in resolving these issues. However, they are familiar with pharmaceutical care and referral criteria, and they are able to identify and refer eligible patients for consultation. “Field diary 31/09/2016.*

Thus, besides less energy required, alongside increased time and continuously updated knowledge, pharmacists could open up to new roles such as implementing pharmaceutical care and performing clinical activities. It is a strategic, continuous, planned and organized process for implementing actions.

#### Contribution to resolution of complex needs

The characteristics of the population attended to at IPGG are: low educational level, low income, polymorbidities, polymedication, care by several professionals and health services, high health service demand, high complexity case management, difficulties for access to drugs through Brazilian health services. Thus, professionals become overwhelmed with numerous and complex activities. The pharmaceutical care service began to respond positively to needs and demands of the staff and older adult population regarding their problems of transition of care through medication reconciliation, which resulted in interventions of pharmaceutical care to external healthcare locations, like basic health units. To optimize the services offered, they expanded pharmaceutical interventions to groups and improve the list of essential drugs, as well as elaborated health care protocols to rationalize and monitor pharmacological treatments offered to eligible patients through individualized care. The complexity of the patients made pharmacists create a pharmaceutical care model that reconciled individual and shared clinical decision-making. In this way, greater health resolution was obtained and pharmacists became recognized as multi-professional team members.

The main reasons for multi-professional team referral to pharmaceutical care service, identified in the documentary analysis (*n* = 224), show that the pharmacist is highly recognized for activities related to promoting treatment adherence (30%). After attending to referral appointments, however, it was discovered that 44% of the main problem this patients were facing was not related to treatment adherence, actually, had other drug-related problems, such as 14% related to indication, 9% related to effectiveness and 21% related to safety. Polypharmacy analysis and monitoring (21.6%) was the second largest cause of referrals by the team, justified by the profile of the users, the polymedicated polymorbid elderly patients. The predominance of referral related to treatment adherence seems to be as a result of consolidated perception, but this is only superficial, of the team regarding the main role of the pharmacist, to guide patients on the appropriate use of drugs. But, even if they are not in numbers, there is this idea among professionals that pharmacists are dedicated to analyze what happens to each patient and that they contribute to the efficient and adequate use of drugs, even though, this is not perceived so clearly. Some excerpts from a talk with professionals illustrate their perspective on the role of the pharmacist:

*“And when they (pharmacists) realize that there is no treatment adherence, and that (the patient) is not taking medication, they evaluate it, mark an appointment for a consultation and then the entire investigation begins.” (Member of the medical team).*

*“It’s an additional help that we have, as I’ve already said, they help in correcting wrong medication, wrong dose and guide patients who are polymedicated”. (Member of the medical team).*

The possibility of the pharmacist solving problems related to drug access was the third cause of referral (18.8%) of patients to pharmaceutical care by professionals of the health team. There are problems of compliance of prescriptions with dispensing rules, guidance on the process and documents necessary for the acquisition of unpaid drugs, possibility of drug acquisition in other places, optimize drug dispensing time, prescription validity and consultation date for new prescription.

“I think one thing that they solve and that should not do much, has to do with the elderly people who without drugs, that happens a lot, they may have missed consultation or the doctor could not attend to them within the deadline to renew the prescription, they resolve this several times.” (Management authority).

In addition to these three main reasons for referral, pharmacists are recognized for their ability to deal with complex situations and the organization of pharmaceutical care, which demonstrates that the pharmacist’s role is consolidated in the institution, since it was reinforced in interview with the multi-professional team, as illustrated in the following passages:

*“We see that you’ve actually taken one more (pill), that one more can hurt. (...). So for me it is something desperate, because I’m not a pharmacist... then I take the case to them. (Member of the multi-professional team).*

*“It is a differentiated service, a more detailed one I think, observing patient by patient, not only distributing drugs, but seeing how it is being administered. They communicate when drugs display adverse effects.” (Member of the multi-professional team).*

#### Organizational visibility

In the IPGG organizational policy, data generation and service production monitoring are required and valued for government account purposes and for financing access actions. According to this institutional culture, pharmacists organized the service by developing reproducible forms and methodologies. Sharing the work with the multi-professional team and the managers generated understanding and acceptance, and thus, referrals were made to the pharmaceutical service.

In a permanent way, the indicators produced by pharmaceutical service are promoted in internal meetings and also communicated in scientific publications, which generates external service visibility to gerontology and pharmaceutical fields. The most commonly used indicators by pharmacists were those related to the results of the service: number of consultations, absences, problems identified, interventions, acceptability of interventions by physicians and other professionals, clinical parameters and clinical improvement. Process indicators, such as time of attendance and referrals were also adopted. This evaluation also responds to the evaluation policy of the institute’s care services:

*“Each process has a goal based on numbers, percentage, quantity. We have goals and what to do to achieve the goals. Then there is a standard operating procedure (SOP) for each target and one responsible for each SOP. After that, we have our schedule for the entire year.”* (Pharmacist 1).

The positive image resulting from this process makes the institute to be highly recognized internally and externally and provokes institutional support to the pharmaceutical care service. Outlining results enables pharmacists to identify strengths, visualize the need for improvements, as well as new opportunities to advance service implementation in a cycle of ongoing evaluation and re-planning.

#### Configuration denied

**Fig. 4:**
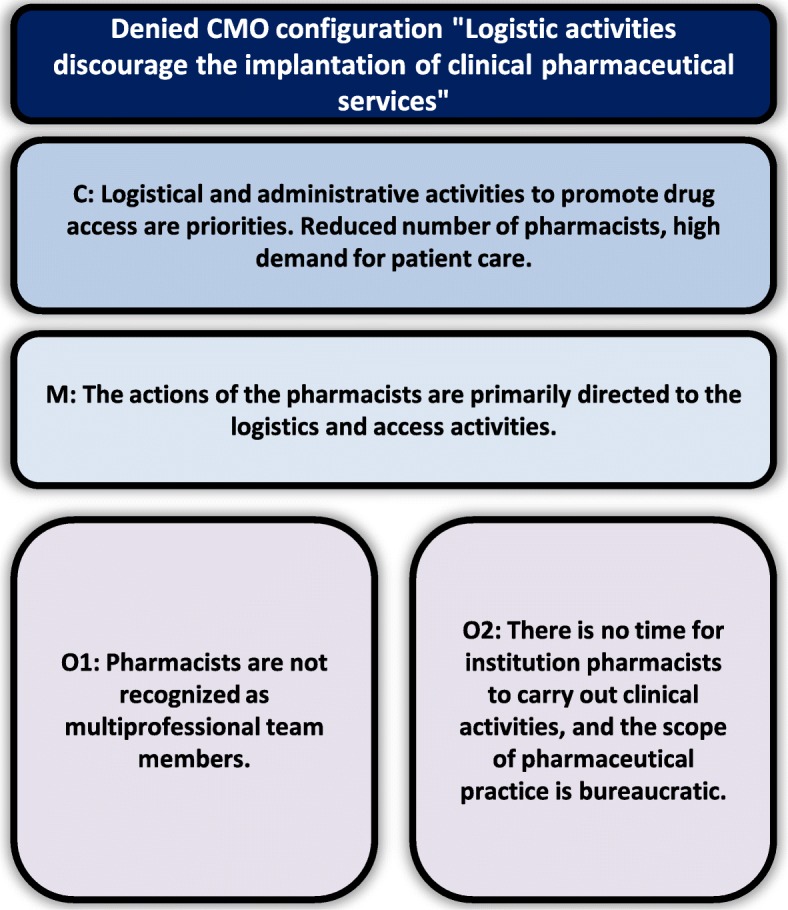
Denied Inter-relationship between C (Context), M (Mechanism) and O (Outcomes) of discourage the implantation of clinical pharmaceutical services by logistic activities

The study denied the configuration “Logistic activities prevent the implantation of clinical pharmaceutical services” (Fig. 4).

The scientific literature indicates that in Brazil and countries with similar health systems or realities, pharmaceutical clinical service implementation is difficult, primarily due to insufficient pharmacists and prioritization of actions related to medication organization and dispensation, with little involvement in care actions and consequently less professional recognition of pharmacist as a member of the health team [48, 49]. This type of difficulty appears in some statements and it was observed in some moments of daily work by participating observation, moments when it turned out that is requiring persistence of pharmacists, who have the understanding that this aspect of their work is indispensable:

“I would love to work only in pharmaceutical consultation, but the demand is another... it is a giant job, is to keep a job of keeping the pharmacy with proper stock.” (Pharmaceutical Services Coordinator).

However, three previously described CMO’s configurations provided interventions for change. An important step was to make a proper diagnosis of the characteristics of the population attended to and their needs. In this way, it was possible to organize pharmaceutical services and access to drugs in a strategic way, thereby reducing problems of access and logistics which usually consume the pharmacist’s time. Pharmacists also improved drug dispensing and inventory management through the implementation of an electronic system which was approved by users and recognized by the multi-professional team. They continue to solve logistical issues, but more focused on strategic and less operational issues, which leaves more time available for pharmaceutical care.

## Discussion

The restriction of the study period in relation to the described period was necessary due to the different forms of pharmaceutical registration adopted in previous or subsequent periods. In this way, uniformity in the description and meaning of the variables and elements studied was ensured.

It would be desirable to increase user participation in the focus group. However, even with this restricted participation and although only one focus group was performed, the participant observation also allowed permanent contact of the main researcher with the users and, consequently the collection of impressions and attitudes of users in the daily life of the service. They also minimize the possible insufficiency of the focus group participants in the triangulation and diversity of research sources, detaily selected according to the objectives of the study.

When considering CMOs configurations to explain the implementation of pharmaceutical service, the performed analysis contributes to understanding the mismatch that may occur between public policies and reality, as pointed out by studies carried out in Brazil and other countries. The interfaces observed in this study reaffirm that health service implementation is a result of multidimensional interactions between the individuals in the context in which they are inserted [50].

One of the central aspects to be considered is the responsibility of providing continuous education for internal and external professionals, aligned with the National Policy of Permanent Education in Health of the Brazilian Ministry of Health guidelines [51, 52]. In this perspective, it is understood that institutions and training centers should work closely with other *SUS* administration agencies in workers continuing education, articulating teaching-service and contributing to changes that make services more adequate and effective [53]. The construction of an environment focused on education and exchange of interprofessional knowledge contributed to engage all those involved in the implementation process, mainly for pharmacists’ motivation to seek more knowledge and practical application. Education and engagement of health professionals meet the needs of the target population and create means to overcome complex barriers [54].

The WHO (World Health Organization) considers polymedicated older adults a risk group, subjected to adverse events and complications, and they need to be submitted to new strategic actions [1]. The complex polypharmacy in older adults leads to the creation of strategic approaches and integration of the pharmacist into the multi-professional team. The objective is to handle polypharmacy situations in order to better results [55, 56]. Effective communication through medication reconciliation at IPGG pharmacy service is integrated with patient safety practice in the transition of patients from the primary to secondary, and hospital care settings.

Health team professionals and IPGG users perceived the importance of pharmaceutical care service and incorporated it into the healthcare process in complex demands, from a non-ideal organizational structure. A pharmaceutical care service that inspires but does not limit itself to theoretical models was produced, characterized by pharmaceutical interventions that seeks the involvement of other individuals to potentialize their actions using shared clinical decision-making [57]. Actions and interventions are not apart, but are articulated approaches to overcome limitations [58], such as the identification of individual’s needs in moments of collective intervention.

The sustainability of this service requires actions that contribute to the diagnosis of failures and achievements, dissemination and continued planning, with an environment favorable to data generation and indicators that leverage organizational changes [30]. Thus, the indicators and goals of the IPGG pharmaceutical service contributed to continuous process establishment and improvements, reassessment and planning; a fundamental step for implementing institutional changes [30]. Also, the internal and external recognition achieved through visibility led to pharmaceutical care service consolidation into standards, documents, actions, and behaviors in the IPGG. This reaffirms that the implementation process is rooted in the organizational culture, as a result it is institutionalized [30].

Similar results, obtained using different analytical methodologies, were verified by Ribeiro M. et al (2018) and Moullin (2016) in studies that assessed the implementation of pharmaceutical care. These point out that the documentation of results and the use of strategies for implementation are fundamental for the success of this service. Gil and collaborators identified in community pharmacies in Spain that the expertise of pharmacists is an important facilitator for the performance of pharmacotherapy follow-up, which our study confirms. Other studies carried out in Australia and the United States reinforce all these aspects by indicating the importance of the multi-professional relationship and of strategic planning for the execution and support of clinical pharmaceutical service [31–34].

The implementation feasibility was a process built on aspects considered impeditive in other services, such as disorganization with respect to drug access [37]. In Brazil, where there is frequent shortage of drugs in public health units, which effectively dispense around 60 to 68% of the prescribed drugs. This is due to problems such as poor management of pharmaceutical care, under-funding and prescribing of drugs that do not appear on lists of reference drugs for these services [59]. So, it is highly valued professional performance that organizes and guarantees medication access. In this sense, drugs provided in a qualified manner and oriented according to client needs were developed in IPGG as an element of professional appreciation.

Strategic logistic needs influenced exclusively and objectively clinical performance, but not similar to that identified in other studies, that considers that logistics activities directly affect the viability of the service [48, 49]. It was observed in the IPGG that, pharmacists have created strategies to deal with logistical procedures based on the involvement of auxiliaries and other professionals, which is consistent with the ideas of Kotter (2012), who believes that, delegating activities is fundamental in overcoming barriers during the implementation process [30]. The pharmacists valued the exchange of knowledge with the employees of the pharmacy, thus increasing the possibility of delegating the execution of some logistical activities autonomously like dispensation of medicines, local organization and ability to solve complex problems of medication access. Thus, the workflows were restructured both in the sense of distribution of logistical activities and the assistants’ understanding concerning the importance of pharmaceutical care, also helping in the identification and referral of patients to the pharmaceutical care service. The results are consistent with those verified by Melo et al. (2017), whereby the prepared assistants were able to identify indicators of problems related to the use of drugs during dispensing, as a result they become participants and active members of pharmaceutical care service implementation [60]. This leads us to realize that considering the number and consistency of patient care and prescriptions, the trained assistants are not only an important part, but are fundamental for the operation, selection and referral of patients to pharmaceutical care service. In this way, pharmacists begin to create more time for clinical activities and appointments. Thus, the training of assistants was a facilitating strategy for the pharmacist to assume new roles and maintain professional integrity, as identified in the excerpts from the interview with the pharmacist in studies of the implementation of pharmaceutical care [35, 49, 61, 62]. There was no pharmacist job loss, but their trajectory and interactions made them members of the team, although they carried out activities different from those they initially imagined doing.

Some factors described in the literature such as the absence of an exclusive place to perform activities and difficulty in accessing clinical documents such as medical records and reports were not difficulties encountered by IPGG professionals [36, 37]. This was because structural adjustments were occurring as the processes were being consolidated [63]. Although structural conditions are considered the fundamental basis for the quality of health services [64] many actions can be developed outside ideal conditions, as in this study, in which it was possible to verify appreciation of opportunities and strategic performance, as Reyes (2014) also verified in her work [65].

There is an essential interaction between the three identified CMO configurations, and the construction of scenarios based on educational actions is the most critical influence that leads to other configurations due to the presence of theoretical models, knowledge production, data evaluation, planning and continuous education.

This relationship was also identified by Ribeiro et al. (2018), who concluded that educational support and the development of service management skills by pharmacists are essential to achieve results and overcome barriers [66]. On this aspect, it is important to point out that, although the educational environment is fundamental for the implementation process, initiatives of universities or other institutes should consider the local autonomy and that of professionals for the continuity of the pharmaceutical care service. Also, viable indicators should not only prioritize the need to generate scientific data, but should actually be applicable in the work routine and useful for dissemination, consolidation and institutionalization of the service. Indicators considered good and adequate by academics who support the structuring of pharmaceutical services are not always perceived as applicable or feasible in the daily routine of the service, this also occurs with standardized methods and instruments [60].

Thus, CMOs articulate themselves cyclically and multi-directionally, denying the CMO identified by a literature review demonstrating that logistic needs impede the accomplishment of pharmaceutical care practice.

This article contributes to the literature by deconstructing some paradigms about performance of pharmaceutical care and health promotion processes commonly seen in other studies. It elucidates and shows for the first time, using realistic evaluation and case study, existing mechanisms for problems that have been questioned for years about the performance pharmaceutical care and promotion of rational use of drugs in Brazil. In addition, demonstrates that the application of implementation science and evidence-based practice is a fundamental way to understand the complexity of pharmaceutical care services.

The analysis from the perspective of realistic evaluation reveals that results are possible in a particular context through mechanisms, and as this was a case study. These results may be valid, especially in similar contextual situations regarding structure, high demand for care and reduced staff; a common situation in Brazil and other countries. However, studies on the implementation of pharmaceutical care conducted in Brazil and other countries show consistent results with those displayed in our study, indicating that the analysis of the CMOs presented in this study may be useful in other implementation scenarios [20, 21, 67, 68].

## Conclusion

Contextual elements led to the adoption of strategic approaches for constructing healthcare scenarios, allowing pharmaceutical care service to contribute to the IPGG care production according to the needs of institute, staff and the target populations. This data reveals the importance of educational processes destined for educational qualification of pharmacists of Brazilian health system, using the transdisciplinary perspective to provide knowledge about the reality of health situation, organization and management, the rational use of drugs, and shared clinical decision-making for health resolution. The present study leads us to reflect if the clinical practice employed during the implementation of pharmaceutical care is consistent with the clinical practice that the context requires and favors. It is possible that the implementation process of pharmaceutical care in different health centers may experience difficulties due to attempts to replicate theoretical models that may disregard complex reality of health systems, especially in developing countries. Thus, mechanisms that seek to identify and work on real needs, favor the generation and sharing of knowledge, collaborative work, reassessment and constant planning which are facilitators of the implementation process of pharmaceutical care services.

## Supplementary information

**Additional file 1.** Semi-structured questionnaire guide for individual interviews and focal group.

## Data Availability

The datasets generated and/or analysed during the current study are available in the Unifesp repository, http://repositorio.unifesp.br/handle/11600/49138 [69].

## Abbreviations

BBS: Bárbara Barros Silva
CEP: Ethics and Research Committee
CF: Claudia Fegadolli
CMO Configurations: Context-Mechanism-Outcome Configurations
GPEMSA / USP-RP: Health Measures Research Group of University of São Paulo - Ribeirão Preto
IPGG: Paulista Institute of Geriatrics and Gerontology
PW: Pharmacotherapy Workup
Qualifar-SUS: SUS National Qualification Program of State Pharmaceutical Assistance Program
SUS: Sistema Único de Saúde
UNESP: State University Paulista Júlio de Mesquita Filho
UNIFESP: Federal University of São Paulo
USA: United States of America
WHO: World Health Organization)
