# Supplementary material for: Implementation of pharmaceutical care for older adults in the brazilian public health system: a case study and realistic evaluation
Source: BMC Health Serv Res. 2020 Jan 14;20:37. doi: 10.1186/s12913-020-4898-z (PMC6958615; doi:10.1186/s12913-020-4898-z)
Supplement: Supplementary file 1 — Additional file 1. Semi-structured questionnaire guide for individual interviews and focal group. [file 12913_2020_4898_MOESM1_ESM.docx]

**Semi-structured questionnaire guide for individual interviews and focal group.**

How the implementation of pharmaceutical care service initiate? (only for pharmacists)

When the implementation of pharmaceutical care service initiate? (only for pharmacists)

Why the implementation of pharmaceutical care service initiate? (only for pharmacists)

In your vision, what is pharmaceutical care?

What do you know about Pharmaceutical Care service in IPGG? Who is involved? (only for staff and patients)

Can you describe the operation of Pharmaceutical Care service in IPGG?

How does the service communicate in IPGG?

How the pharmaceutical services contribute to health care in IPGG?

Why the existence of Pharmaceutical Care service in IPGG is important?

What challenges does the service need to face to improve its processes and results?

Which elements are favorable to the existence and maintenance of the service? (only for pharmacists)

Which elements make it difficult to exist and maintain the service? (only for pharmacists)

How pharmaceutical care service helps you? (only for patients)

Do you want to say anything else that I didn't ask?
